# Transcranial Magnetic Stimulation Studies of Sensorimotor Networks in Tourette Syndrome

**DOI:** 10.3233/BEN-120289

**Published:** 2013

**Authors:** Michael Orth, Alexander Münchau

**Affiliations:** ^1^Department of NeurologyUniversity of UlmUlmGermany; ^2^Department of NeurologyUniversity Medical Center Hamburg EppendorfMartinistrHamburgGermany

**Keywords:** Transcranial magnetic stimulation, axon, synapse, sensori-motor integration

## Abstract

Gilles de la Tourette syndrome (GTS) is a sensorimotor disorder where the sensitivity to external and internal stimuli might be increased and unwanted responses to such stimuli cannot be sufficiently suppressed.

Transcranial magnetic stimulation (TMS) studies indicate that, at rest, axonal excitability of cortico-spinal neurons and intra-cortical inter-neurons was consistently normal in GTS. However, synaptic excitability in cortico-spinal neurons and the SICI circuit may be lower than normal. In addition, an electrophysiological marker of sensory motor integration, SAI, was reduced in the baseline state consistent with reduced efficiency of synaptic inhibition. Given the possible influence of sensory inputs in triggering the release of tics reduced SAI may be a direct physiological reflection of increased access of sensory input to motor output in GTS. Experiments examining control of voluntary movements revealed that in GTS motor cortex excitability increases less than in controls when preparing a movement even though intra-cortical inhibition (i.e. SICI) normalises.

In GTS the gain of many motor circuits may be reduced and hence less sensitive to small changes in input from other areas. These cortical changes may constitute an adaptive response to abnormal basal ganglia-motor cortex inputs.

## 1. Introduction

Chronic motor and phonic tics with childhood onset define Gilles de la Tourette syndrome (GTS). Simple tics often relieve internal sensory urges felt in the area of the tic (premonitory sensations) [1] while more complex tics such as echophenomena respond to the perception of external stimuli. Environmental factors, such as emotions or stressful situations, may also alleviate or exacerbate tics. These clinical observations suggest that GTS is a sensorimotor disorder where the sensitivity to external and internal stimuli might be increased and unwanted responses to sensory, motor or emotional stimuli cannot be sufficiently suppressed [2, 3]. This may lead to tics and maybe other habitual motor responses of, or associated with, GTS such as forced touching or compulsions [4].

Pathophysiologically, the origins of tics very likely involve abnormal processing in basal ganglia-thalamo-cortical circuits in which information from many sources needs to be integrated with motor output. A glitch in this complex process may provide the driving force of a chain of events that culminates in unwanted behaviour such as tics over which patients have incomplete control [5]. Only a few brains of patients with GTS have been studied post-mortem so that the understanding of the neurobiological underpinnings of tics has to rely largely on *in vivo* studies [6]. Different methods help investigate brain structure, such as magnetic resonance imaging (MRI). This is complemented by functional analyses using new techniques such as functional MRI (fMRI), which allows studying brain function *in vivo*, including areas deep within the brain including the basal ganglia. FMRI assesses task related changes in blood flow. An increase in blood flow equals an increase in brain activity. The type of ac-

ISSN 0953-4180/13/$27.50 © 2013 – IOS Press and the authors. All rights reserved tivity though remains uncertain. For instance, both inhibitory and facilitatory activity may give rise to similar changes in blood flow. In contrast, electrophysiological methods can differentiate the quality of brain activation and can therefore add an important dimension to the non-invasive study of brain function.

In this review, first transcranial magnetic stimulation is briefly introduced as a method before the data from studies with GTS patients are reviewed. For some time, studies have focused on tics or when patients were at rest; more recently, this has been extended to the physiology of voluntary movement control. Therefore, the focus is first on what is known about the electrophysiology of tics and the resting state before moving on to voluntary movement control, i.e. the preparation, execution and inhibition of voluntary movements. Next, repetitive transcranial magnetic stimulation as a treatment option is discussed. The review closes with a synthesis of current electrophysiological thinking including a number of unanswered questions.

### *1.1. Transcranial magnetic stimulation*

Transcranial magnetic stimulation (TMS) takes advantage of the principle of induction. Differently shaped coils of wire (e.g. round, figure-of-eight) encased in plastic are placed on the head. The coils are connected to a rapidly discharging large capacitator, which sends a strong current of about 8000 A for about 1 ms through the windings of the induction coil. This in turn generates a magnetic field that is oriented perpendicularly to the plane of the coil. The magnetic field passes unimpeded through the skin and skull and induces a directed current on the surface of the brain that flows tangentially to the skull. If high enough this current depolarises nearby nerve cells in much the same way as if currents were applied directly to the cortical surface. Transcranial electrical stimulation (TES) of the brain had been used before TMS. However, electrical stimulation given to the scalp can be painful and is thus limited by the discomfort it produces. TMS has the advantage that the electrical current is induced in the brain and is therefore painless. Also, the activity of intracortical inhibitory and facilitatory circuits can be delineated more reliably with TMS than TES. TMS is safe if the protocols adhere to the safety guidelines [7] so that it can be used in awake human beings.

Using TMS, there are currently two main ways of stimulation. Single or paired pulse TMS can depolarise populations of neurons in the neocortex resulting in an action potential. The effect of TMS depends on the coil position over the brain. If the coil is placed over the motor cortex TMS induces a discharge in pyramidal cells and/ or intracortical interneurons projecting onto them. This leads to a volley that descends the pyramidal tracts in the brain and spinal cord, and then the peripheral nerve. The peripheral nerve makes contact with a muscle; upon arrival of the descending volley a muscle twitch is visible and can be recorded as a motor-evoked potential (MEP). TMS can produce MEPs in various individual muscles or groups of muscles depending where in the motor cortex the electrical current is induced. Commonly, one induces a twitch in a small hand muscle, e.g. the first dorsal interosseus (FDI). These effects do not outlast the period of stimulation.

Trains of stimuli can also be given with TMS (repetitive TMS or rTMS) (for a review see [8,9]), or such trains can consist of paired stimuli with one stimulus given to a peripheral nerve and the other to the motor cortex (paired associative stimulation [10]). Depending on the frequency and intensity of stimulation, and coil orientation, rTMS can increase or decrease the excitability of cortico-spinal or cortico-cortical pathways. In contrast to single, or paired pulse TMS, these effects can outlast the period of stimulation (usually for about 20–30 minutes). The participant usually does not consciously notice any effects; however, there may be subtle changes of behaviour or brain activity similar to single pulse techniques described above [11].

## 2. Studies using transcranial magnetic stimulation

### *2.1. Cortico-spinal motor neurons at rest*

TS patients do not always tic. Tics tend to occur in bouts with these bouts also occurring in bouts and so on (bouts-of-bouts-of-bouts). There are truly tic-free intervals. There may also be times when GTS patients have no obvious tics but are not free of sensory urges even though they can be resisted at these times. This indicates that the neural circuits driving tics, or helping to resist them, including the motor cortex are not always in the same state. Several studies [3,12] have shown that the intensity needed to induce an MEP (motor threshold) at rest and in a pre-activated state was similar in GTS patients and controls. However, with higher stimulation intensity, GTS patients recruited considerably fewer cortico-spinal neurons compared with controls [13].

Threshold depends on the excitability of axon membranes at the site of stimulation and the membrane potential of post-synaptic neurons in motor cortex and spinal cord. When actively contracting a muscle, synaptic excitability is high so that changes in threshold usually are thought to reflect changes in axonal excitability. Given that active threshold was similar in GTS and controls axonal excitability is probably normal in these patients. With muscles in a relaxed, or resting, state, synaptic excitability is less well specified than when active. Nevertheless, equal resting thresholds in patients and healthy controls suggest that the most excitable connections (i.e. those recruited at threshold) are in the same state in both groups [3,12–14].

Above threshold, further neurons are recruited so that the resulting MEP becomes larger. The slope of MEP recruitment depends on how excitable the neuronal elements in the cortico-spinal system are that need higher intensities of stimulation than those activated at threshold. If there were little difference between the excitability of the most and least excitable members of this population, then a small increase in stimulus intensity would recruit many additional connections and create a large MEP: the gain of the input-output slope would be steep. In contrast, if the difference in excitability within the cortico-spinal neuron population were greater, then the same change in intensity would recruit only a small number of extra connections and the MEP would remain small: the input-output relation would be shallow. Since GTS patients recruited MEPs more gradually there seems to be a greater difference between the most and the least excitable cortico-spinal neuron than normal [13]. It is interesting to note that in a more recent study the recruitment slopes of controls and GTS patients were similar at rest [14]. Assuming that this was not caused by a difference in the controls this is not easily explained. One possibility is that the two GTS cohorts were clinically different.

In fact, GTS patients in the Orth et al. cohort were more severely affected than those in the Heise cohort. It is well known that cortico-spinal excitability decreases when movements are inhibited (for a review see [15], and see below). Thus, in patients with more tics there may be a greater need to inhibit movements in response to e.g. sensory urges even though no movements are apparent during the experiments. If that were the explanation for the reduced cortico-spinal excitability then one might want to speculate that those GTS patients were in a state of (tic) movement inhibition rather than truly at rest. However, an argument against this assumption would be that with movement inhibition, at least in healthy controls, short-interval cortical inhibition (SICI) probably increases. In GTS, SICI is decreased (see below).

### *2.2. Inhibitory and facilitatory circuits at rest*

Several TMS studies in GTS have demonstrated reduced short-interval cortical inhibition (SICI) at rest [3, 13,14,16]. SICI and intra-cortical facilitation (ICF) reflect the activity of intra-cortical inhibitory and facilitatory inter-neurons. These inter-neurons contribute to the regulation of the excitability of cortico-spinal neurons within the motor cortex. In the SICI circuit the threshold intensity needed to produce SICI and the amount of SICI at supra-threshold intensities of conditioning shock can be distinguished [17]. GTS patients have normal thresholds for SICI, but recruitment of inhibition at supra-threshold intensities is reduced [12, 13]. Similar to the cortico-spinal neurons it is thought that TMS pulses recruit SICI by exciting axons and that this then leads to synaptic release of inhibitory neurotransmitters such as GABA. Thus, normal thresholds in the presence of decreased recruitment would be compatible with the idea that in GTS axonal excitability is normal whereas the recruitment of synaptic inhibition in the SICI circuit is reduced. GTS patients as a whole had increased ICF in one study [18], whereas others had not described any significant effects [2,3,19]. This could simply be because of the well-recognised variability in measurements of ICF [17] but may also relate to varying intensities of the conditioning stimulus, the number of patients studied and/or their co-morbidities.

A further inhibitory circuit can be examined when a TMS pulse is given to the motor cortex during tonic contraction of a target muscle; this produces a motor evoked potential (MEP) followed by a period of EMG silence before the activity resumes its pre-stimulus baseline level of activity. This is referred to as cortical silent period (CSP). CSP duration may be shorter in patients than in controls [3,12], or normal [20], especially when corrected for differences in absolute MEP amplitude between patients and controls [13]. Thus, the relation between cortico-spinal excitability and the inhibitory mechanism responsible for the CSP – presumably through cortico-spinal motor neuron recurrent collaterals [21] – is the same in patients and controls. This supports the notion that not all inhibitory systems are affected in GTS. The above provides good evidence to suggest that the cortico-spinal system and the inter-neuron circuits that shape cortico-spinal output have in common a reduction of synaptic excitability.

Communication of cognitive, motor and sensory information between the hemispheres involves the corpus callosum. Inter-hemispheric inhibition between the motor cortices can also be investigated with TMS [22].

In a sample of pure, un-medicated, GTS patients left to right inhibition was weaker than right to left, and left to right inhibition also differed to controls [23]. The asymmetry of inter-hemispheric inhibition was thus the opposite of what has been described by the same authors in normal controls [24]. Whether this reflects an abnormal development of these inter-hemispheric connections remains to be seen. However, in the same patients imaging revealed no evidence for any structural differences in the motor region of the corpus callosum [23]. The interpretation of these findings remains difficult; however, this illustrates that brain function and brain structure can be altered independently.

### 2.3. Sensory-motor integration at rest

It is often crucial, e.g. to avoid injury, that sensory input, e.g. pain, translates into movement. Sensory afferent information travelling in sensory-afferent pathways (peripheral nerve, spinal cord, thalamus, sensory cortex) therefore needs to be linked to motor output. If some tics are an unsuppressed response to sensory urges, then we might expect to see abnormalities in inhibitory pathways that specifically link sensory input and motor output. Abnormal sensory motor gating has been proposed in studies assessing grip force control. GTS patients employed higher grip forces to manipulate an object while they were able to adjust grip force similar to controls when the load force changed [25,26]. Interestingly, the sensory-motor cortex has also been implicated in the generation of tics in children [27].

One sensory motor integration pathway that can be tested in humans is sensory afferent inhibition (SAI: [28]). A transient sensory input (electrical pulse given to the median nerve at the wrist or terminal sensory afferents at the fingers) leads to a rapid and short lasting motor cortex inhibition. SAI was reduced in the baseline state in patients [12] consistent with the assumption of a reduced efficiency of synaptic inhibition. Given the possible influence of sensory inputs in triggering the release of tics reduced SAI may be a direct physiological reflection of increased access of sensory input to motor output in GTS. It is interesting that in GTS patients, but not in controls, a single dose of nicotine was able to strengthen the inhibitory effect measured with SAI and also SICI [16]. This suggests that cholinergic stimulation can modulate the circuits underlying SAI and SICI.

### 2.4. Association of TMS data with tic ratings

If experimental data relate to the cause of the clinical phenotype of GTS, i.e. motor and phonic tics, one would expect an association between the two. An association alone would, of course, not prove a causal relationship. Ziemann and colleagues showed that patients with distal tics, i.e. hand tics, and those not taking dopamine receptor antagonists had shorter cortical silent period duration and less SICI [3]. In addition, Gilbert et al. demonstrated that less SICI was associated with greater motor tic severity as measured with rating scales, in particular in those patients that did not take dopamine receptor antagonists and those that had co-morbid attention deficit hyperactivity disorder (ADHD) [20]. In untreated patients, we measured tic severity and distribution with standard clinical scales as well as detailed video analysis and correlated these with recruitment of cortico-spinal output and silent period duration elicited by TMS [13]. Our hypothesis was that clinically meaningful changes in cortical excitability would be specific to those tics that involve the cortical areas examined with TMS, e.g. hand and finger tics. Input-output measures were correlated with video tic ratings for severity of complex tics, phonic tics and hand/finger tics while other tic ratings, i.e. scores on the Yale Global Tic Severity Scale (YGTSS [29]), the Modified Rush Video Scale [30] or other raw tic video scores, were not. Increased excitability of the motor cortex at rest was associated with more frequent tics. Predictions of complex tic, hand and finger tic and phonic tic frequency were only significant in the subgroup of patients without co-morbidity. Comorbidity such as ADHD very likely influences cortical excitability [20] with evidence suggesting that abnormalities in GTS+ADHD may be more widespread than in GTS alone [18]; therefore co-morbidity may have introduced noise into the recordings from the whole group of patients. Complex tics likely involve a large part of the motor cortex including the M1 hand area thus explaining the specific association of complex tic and hand/finger tic frequency with TMS measures from that area. Therefore, the specific association of electrophysiological measures from the M1 hand area of the motor cortex with movements originating from the same area, i.e. hand/finger tics in contrast to other body areas, suggests the difference in the excitability of the motor-neuron pool in a resting state could be clinically meaningful. In contrast, in line with motor cortex excitability levels in a pre-activated state being similar to normal controls there was no association of these measures with tic ratings.

Interestingly, Heise and colleagues observed an association of tic severity with the ability to normalize SICI when preparing a movement [14], see below.

### 2.5. Voluntary movement control

Movement is an integral part of human life. Movements need to be selected, planned and executed. In addition, one needs to get the timing right so a movement has to be withheld, i.e. inhibited until the right moment arrives. These complex tasks predominantly involve the pre-frontal cortex, the basal ganglia, the supplementary motor area (SMA) and the pre-SMA with subsequent integration of these inputs in the primary motor cortex (M1). In GTS, the unwanted movements and their sensory prodrome add to this already complex process. Patients report that their tics, or the sensory urges, may interfere with planned movements or speech.

On the other hand, it is a well-recognized phenomenon that engaging in voluntary activity requiring attention such as playing an instrument can virtually abolish sensory urges and tics [13]. This illustrates that the forces driving tics compete with physiological fronto-striatal network activity.

Changes of the excitability of the motor cortex before a planned movement, or when movements have to be withheld, can be measured using TMS. In reaction time paradigms involving Go-NoGo tasks, a reduction of intra-cortical inhibition precedes an increase in cortico-spinal excitability (larger MEP amplitude)(for a review see [15]). Cortico-spinal excitability increases gradually from about 100 ms to 40 ms prior to muscle activation and then much sharper from 40 ms to the actual movement (EMG activity) [31]. Reduced SICI and increased cortico-spinal excitability are maintained until about 50 ms before the end of muscle activation when both measures return to their baseline excitability levels [32,33]. Motor cortex excitability may already change in a similar way when the need for a movement is imminent (expectation of an imperative cue in a reaction time task [34]). In contrast, cortico-spinal excitability decreases when a movement needs to be prevented [35,36].

During movement preparation cortical excitability increased less in GTS compared to in controls [14]. On the other hand, SICI normalized quickly from reduced levels at rest (see above) [14]. This increase in intra-cortical inhibition early on during movement preparation was particularly marked in those patients with fewer tics in whom cortico-spinal excitability was also lower. This suggests that tic control depends on the extent to which excitability of motor neurons can be reduced in line with an increase in the activity of intra-cortical inter-neurons.

### 2.6. Repetitive transcranial magnetic stimulation

Imaging studies revealed that during tic suppression and tics, activity was increased in pre-motor, pre-frontal and motor cortex [37–39]. This led to the hypothesis that normalising motor or pre-motor cortical excitability might be a potential tic treatment. Low frequency repetitive transcranial magnetic stimulation (rTMS) has inhibitory effects on the stimulated brain area [40]. This lead to the rationale for small treatment trials using 1 Hz rTMS with an intensity of 80% active motor threshold to inhibit the pre-motor or motor cortex [41, 42]. These trials included a sham (i.e. placebo) condition, and the effects on tics were evaluated using a clinician-rated tic scale (YGTSS) [29], video ratings and self-ratings. No effects on tic severity were noted; even with higher stimulation intensities and when given over longer time periods tics did not improve consistently in GTS in a further sham-controlled trial [43]. These results do not support a role for motor or low intensity 1Hz pre-motor rTMS in the treatment of tics in GTS. Subsequently, Montavani and colleagues reported that a different rTMS protocol using higher intensities of stimulation (100% resting motor threshold, bilateral SMA) given over ten days had a beneficial effect on tics, OCD and other measures that lasted for three months [44]. Similar effects were reported by Kwon and colleagues in 10 boys treated with 1Hz rTMS at 100% RMT over the SMA [45]. RTMS may therefore have some promise as a potential treatment; however, the latter studies did not include a sham condition (i.e. placebo control) so it remains unclear how much of the effects were actually due to a placebo effect.

Recently, rTMS protocols have been used to examine plasticity in GTS. Two studies observed no plasticity effects of inhibitory or facilitatory theta burst rTMS in motor cortex, or brainstem, in GTS patients [46,47]. This suggests abnormal plasticity in GTS. However, the effects of rTMS depend on several factors including the state of the stimulated brain area before, during and after stimulation, or medication [9]. Activity can have quite pronounced effects on the direction of plasticity effects. To induce the desired effects it is important that the participant is at rest before and during stimulation. The absence of any visible movement may not be synonymous with the motor cortex being at rest [48]. Thus, the motor cortex may be quite busy, e.g. dealing with sensory urges, even though the patient has no obvious movements. Therefore, the results of experiments addressing plasticity have to be interpreted with caution.

## 3. How specific are the findings to Tourette syndrome?

The electrophysiological findings described above are not specific to GTS. Similarly altered motor cortex excitability has been found in other movement disorders, such as Parkinson's disease, atypical parkinsonian syndromes, or dystonia (for a review see [49]). In Huntington's disease, reduced SAI and flatter input-output curves have been observed; SAI might be a state marker as it changed with proximity to motor onset while input-output curves did not and might thus represent a trait marker of carrying the HTT repeat expansion mutation [50]. It is thus clear that TMS paradigms cannot aid in the differential diagnosis of these movement disorders. However, they may have added value as biomarkers, e.g. in Huntington's disease. In GTS, they may help delineating endophenotypes and thus be useful for the search of underlying genetic variants.

## 4. Conclusions and perspectives for future research

What matters most to patients with GTS is how bad their tics are and how much control they have over them. This reflects the balance between underlying deficits and adaptive, compensatory changes in different parts of cortico-subcortical networks involving the basal ganglia, motor and pre-motor cortex, thalamus and pre-frontal cortex. Much as tics take a waxing and waning course in intensity, and can occur in bouts, the interactions between different parts of these cortico-subcortical networks, and ultimately their influence on shaping motor output, are not static but change continuously.

Bearing this in mind, in the resting state axonal excitability of cortico-spinal neurons and intra-cortical inter-neurons was consistently normal in GTS. However, there is evidence that synaptic excitability in cortico-spinal neurons and the SICI circuit is lower than normal. In addition, an electrophysiological marker of sensory motor integration, SAI, was reduced in the baseline state consistent with the assumption of a reduced efficiency of synaptic inhibition. Given the possible influence of sensory inputs in triggering the release of tics reduced SAI may be a direct physiological reflection of increased access of sensory input to motor output in GTS. When reduced SICI was first noted in GTS, it was conceivable that reduced inhibition in the cortex meant the motor cortex was more likely to release involuntary movements in GTS. However, more recent evidence indicates that the gain of many motor circuits may be reduced in GTS, with the result that they become less sensitive to small changes in input from other areas. The more effectively the motor cortex can reduce its excitability the more effectively tics can be controlled. Cortical inhibition may thus mirror an adaptive response to abnormal basal ganglia-motor cortex inputs that prevents the release of unwanted movements unless input signals are strong enough to overcome the reduced processing gain. A caveat to this interpretation is that, in the authors' experience, many GTS patients who take part in research can sit still for the time the experiments take. This does not necessarily mean they are free of symptoms; sensory urges may still be present so that the situation in the laboratory may model a state of enhanced tic suppression. The data recorded in this state may well resemble those recorded when a normal movement is prepared but then has to be actively suppressed. Experiments examining control of voluntary movements revealed that in GTS motor cortex excitability increases less than in controls when preparing a movement even though intra-cortical inhibition (i.e. SICI) normalizes. At the same time suppression of movement is associated with more wide-spread cortical activity in GTS (increased cortico-cortical coupling on EEG- and MEG-coherence analysis) [51,52].

It is interesting to speculate that GTS patients may spend much more time than controls in a state of movement preparation because of tic-driving forces; they may be able to then suppress these movements so from the outside they seem at rest. To the researcher in the laboratory the challenge would be to distinguish these states because the motor cortex can be quite active without any apparent movements [48].

A key challenge remains the distinction between what may be a primary abnormality, which hence contributes to causing tics, and what may reflect secondary, e.g. compensatory, changes. It is difficult to draw any conclusions from cross-sectional designs, and longitudinal studies may take a long time and are also difficult in a disorder that does not per se progress in adulthood. A further important question is how the circuits relevant for movement control and sensory gating develop from childhood to adulthood. The occurrence of tics in childhood suggests GTS is a developmental disorder. Some tics are present for a short period of time in many healthy children as well but then disappear. This suggests that tics persist and GTS thus develops when the neuronal circuits responsible for sensory gating and movement control do not mature appropriately. It is interesting to note in this regard that SICI was reduced in the motor cortex in healthy children, and to some degree in adolescents, while intra-cortical facilitation and conduction times along the cortico-spinal tract were normal [53,54]. While being cautious to extrapolate from cross-sectional data this may mean that reduced intra-cortical inhibition is necessary for motor learning in childhood and adolescence. Reduced SICI in GTS could then be a sign of abnormal maturation of the motor brain.

The combination of a variety of techniques such as EEG, MEG, TMS and imaging may also be helpful to inform us about effects at a network level after a well-defined challenge to the motor cortex. Such complementary assessments have already proven useful for the understanding of brain function [11]. Some of the above techniques capture activity of the whole brain while TMS measures activity within the confines of the motor cortex or can manipulate excitability in that region. One could, for example, use one of the repetitive TMS protocols to manipulate motor cortex excitability and evaluate its effects on neuronal plasticity not only at the local level with TMS but also at the network level using imaging techniques. Recording from electrodes implanted for deep brain stimulation, so far an experimental new form of treatment for GTS (e.g. [55]) may also be valuable in this regard similar to Parkinson's disease [56].
